# Anti-matrix metalloproteinase-9 DNAzyme decreases tumor growth in the MMTV-PyMT mouse model of breast cancer

**DOI:** 10.1186/bcr3385

**Published:** 2013-02-13

**Authors:** Miranda A Hallett, Bin Teng, Hisashi Hasegawa, Luciana P Schwab, Tiffany N Seagroves, Tayebeh Pourmotabbed

**Affiliations:** 1 Department of Microbiology, Immunology and Biochemistry, University of Tennessee Health Science Center, 858 Madison Ave., Memphis, TN 38163, USA; 2 Center for Cancer Research, Department of Pathology, University of Tennessee Health Science Center, 19 S. Manassas St., Memphis TN 38163, USA

## Abstract

**Introduction:**

Despite continued improvements in diagnosis, surgical techniques, and chemotherapy, breast cancer patients are still overcome by cancer metastasis. Tumor cell proliferation, invasion and metastasis are mediated, at least in part, through degradation of basement membrane by neutral matrix metalloproteinases (MMP) produced by tumor and stromal cells. Evidence suggests that MMP-9 plays a significant role in breast tumor cell invasion and metastasis. DNAzymes or catalytic oligonucleotides are new classes of gene targeting molecules that bind and cleave a specific mRNA, resulting in decreased protein expression.

**Methods:**

The application of anti-MMP-9 DNAzyme (AM9D) for the treatment of primary and metastatic breast cancer was evaluated *in vitro *and *in vivo *using MDA-MB-231 cells and the MMTV-PyMT transgenic breast cancer mouse model. Spontaneously developed mammary tumors in MMTV-PyMT transgenic mice were treated intratumorally with naked AM9D, once a week for 4 weeks. The stability of DNAzyme was determined *in vitro *and *in vivo *using fluorescently labeled DNAzyme.

**Results:**

AM9D specifically inhibited expression of MMP-9 in MDA-MB-231 cells resulting in reduced invasive property of these cells by 43%. Weekly intratumoral treatment of spontaneously developed mammary tumors in MMTV-PyMT transgenic mice was sufficient to significantly reduce the rate of tumor growth and final tumor load in a dose dependent and statistically significant manner (*P *< 0.05). This decrease in tumor growth was correlated with decreased MMP-9 protein production within the treated tumor tissues. Tumors treated with AM9D were also less vascularized and contained more apoptotic cells compared to control and untreated tumors.

**Conclusions:**

These results show that targeting and down regulation of MMP-9 by AM9D could prove useful as a therapy against breast carcinoma tumor growth and invasion.

## Introduction

Breast cancer is one of the leading causes of cancer death in women, second only to lung cancer [1-3]. The majority of morbidity and mortality amongst cancer patients is due to metastasis of tumor cells to distant organs [2,4]. Breast cancer most commonly metastasizes to bone, lymph nodes, lung, liver, and brain [5]. Despite continued improvements in diagnosis, surgical techniques, and chemotherapy, lethality from breast cancer remains high.

Matrix metalloproteinase-9 (MMP-9) production by tumor and stromal cells is one of the most important factors for metastatic behavior of tumor cells [6-8]. MMP-9 is a member of the metzincin family of enzymes, which play an important role in normal physiological responses, including wound healing and bone formation [9]. MMP-9 becomes deregulated during tumorigenesis and is associated with pro-oncogenic events such as neo-angiogenesis, tumor cell proliferation and metastasis [10]. High level of MMP-9 expression in breast cancer is positively correlated with enhanced tumor cell invasion and metastasis [11,12] and with enhanced progression and poorer prognosis [10].

MMP-9 is conserved across several species (human, chimpanzee, dog, cow, mouse, rat, chicken, zebrafish, and *Arabidopsis thaliana*). MMP-9 degrades type IV collagen, one of the most abundant collagens in the extracellular matrix (ECM) [13], which may stimulate local invasion, the first step in metastasis. In addition, MMP-9 also cleaves pro-cytokines, chemokines, and growth factors, thereby modifying their biological activity [14-16]. The downregulation of MMP-9 has been shown to increase β1-integrin expression, leading to activation of extracellular signal-regulated kinases (ERKs) and increasing apoptosis through one of two mechanisms: (1) release of cytochrome C into the cytosol and/or (2) increase in nuclear factor-κB (NF-κB) activation, followed by activation of caspase-3 [17]. Although few normal cell types express MMP-9 under normal physiological conditions, the majority of human metastatic tumor cells that have been tested consistently show elevated MMP-9 activity compared with benign control cells, including melanoma, fibrosarcoma, breast adenocarcinoma, and glioma [18-21]. In addition, tumor cells that stably express MMP-9 cDNA have been shown to have enhanced metastastic ability [22]. Thus, inhibition of MMP-9 expression could be a useful therapeutic modality to decrease the growth and invasive properties of tumor cells.

RNA-cleaving phosphodiester-linked DNA based enzymes (DNAzymes) are catalytic DNA molecules that specifically bind to and cleave targeted mRNA in a sequence-specific manner. The result is efficient degradation of the mRNA transcript, and thus, similar decreased expression levels of the encoded protein [23,24]. Catalytic oligonucleotides have emerged as novel, highly selective inhibitors or modulators of gene expression [25]. Khachigian and colleagues have reported that the DNAzymes targeting early growth response factor-1 (*Egr1*) mRNA inhibit neointimal formation after balloon injury to the rat carotid artery wall and reduce intimal thickening after stenting of pig coronary arteries [26]. DNAzyme targeting c-Jun causes repair of injured carotid arteries in rats [27]. Finally, a DNAzyme targeting vascular endothelial growth factor receptor 2 (VEGFR2) significantly inhibits the growth of breast tumors derived from xenografting of MDA-MB-435 cells into nude mice by inducing apoptosis [28].

Here, we examine the effects of a novel anti-MMP9 DNAzyme (AM9D) on breast tumor growth in the mouse mammary tumor virus-driven polyoma virus middle T oncoprotein transgenic (MMTV-PyMT) mouse model of breast cancer. We demonstrate for the first time that once-weekly intratumoral injection of AM9D in the absence of any carrier molecule, for four weeks, was sufficient to significantly reduce the rate of tumor growth and final tumor load in a dose-dependent and statistically significant manner (*P *≤0.05). Together, the data presented here justify the further development of AM9D for its potential as an anti-tumor agent and as an ideal candidate for breast cancer therapy.

## Materials and methods

### DNAzyme

All DNA oligonucleotides used in these experiments were synthesised by Integrated DNA Technology (Coralville, IA, USA). DNAzymes were designed according to the specific rule of 10-23 DNAzyme [29]. The DNAzyme targeting *MMP9 *mRNA contains a catalytic domain of 15 highly conserved deoxynucleotides flanked by two substrate-recognition domains. The sequence of the DNAzyme targeting mRNA of mouse and human MMP-9 is 5'-GTGGTGCCAGGCTAGC TACAACGATTGAGGTCG-3'. In the control DNAzyme, 5'-CTAGTCAGCGGCTAGCTACAACGATAAGCTGCT-3', the catalytic sequence of DNAzyme is flanked by nine bases randomly chosen and not specific for any MMP coding sequence. In some cases, the DNAzyme was end-labeled with Alexa Fluora C5-melamide 633 or Oregon Green™ 488 C5-maleimide (Invitrogen, Carlsbad, CA, USA) using T4 Polynucleotide kinase, as suggested by the manufacturer's protocol.

### Cell transfection

MDA-MB-231 human breast tumor cell lines (ATCC, Manassas, VA, USA) were plated in DMEM supplemented with 10% fetal bovine serum (FBS) and allowed to grow to 80 to 90% confluence at 37°C with 5% CO_2_. The cells were then serum-starved for 4 hours prior to transient transfection with Oregon Green™488-maleimide-labeled AM9D or control DNAzyme (24 μg) using Lipofectamine 2000 (Invitrogen). After 18 hours incubation at 37°C in serum-free medium, cells were collected and sorted, and the transfected cells were isolated for further analysis.

### Analysis of *MMP9*, *MMP1*, *MMP13*, *MMP14*, *MMP19 *and *MMP21 *mRNA levels in transfected cells

The *MMP9*, *MMP1*, *MMP13*, *MMP14*, *MMP19 *and *MMP21 *mRNA expression levels in the DNAzyme-transfected cells were quantified by reverse transcription-polymerase chain reaction (RT-PCR) using specific *MMP9 *(forward primer; 5-GCAGGAATGCGGCTCTGG-3', reverse primer; 5'-CCCGTCGAAGGGATACC-3'), *MMP1 *(forward primer; 5'-CATTCTACTGATATCGG-3', reverse primer; 5'-AGAAAACAGAAATGAAA-3'), *MMP13 *(forward primer; 5-GAC TTCCCAGGAATTGGTGA-3, reverse primer; 5-TGA CGCGAACAATACGGTTA-3'), *MMP14 *(forward primer; 5'-GAGCTCAGGGCAGTGGATAG -3', reverse prime; 5'- CCACCTCAATGATGATCACC -3'), *MMP19 *(forward primer; 5'-GGGTCCTGTTCTTCCTACAT-3', reverse primer; 5 CAATCCTGCAGTACTGGTCT-3'), and *MMP21 *(forward primer; 5'-AACAATAGGACACGCTATGG-3', reverse primer; 5'-CATCTCTTTTCCATGTCCAG-3') primers [30]. Total RNA from the transfected cells was isolated by Trizol reagent (Invitrogen) and reverse-transcribed with random hexamer primers (Promega, Madison, WI, USA) using MMLV-RT enzyme (Invitrogen, Carlsbad, CA).

Mouse or human *BACT *(β-actin) mRNA was also amplified as internal controls, with corresponding (human forward; 5'-CAAGAGATGGCCACGGCGGCT-3', human reverse; 5'-TCCTTCTGCATCCTGTCAGCA-3', mouse forward; 5'-CAGGAGATGGCCACTGCCGCA-3', mouse reverse; 5'-AAGCACTTGCGGTGCACGATG-3') primers. The PCR products were subjected to 2% agarose gel and visualized by ethidium bromide staining. Expression was quantified by an Alpha Imager 2000 documentation and analysis system (Alpha Innotech Corporation, San Leandro, CA, USA).

### Analysis of MMP-9 activity by gelatin gel zymography

MDA-MB-231 cells were transiently transfected with AM9D or control DNAzyme in serum-free medium as stated above. Twenty-four hours post transfection-media were collected and concentrated 10-fold using Amicon Ultracell filtration units (Millipore, Co Cork, Ireland). Protein concentration of the collected media was determined by Bradford dye binding techniques (a standard Bio Rad assay) using bovine serum albumin as a standard. The MMP-9 activity in the culture media was then assessed by gelatin zymography [31].

### Cell invasion assay

Cells were transfected with fluorescently labeled AM9D or control DNAzyme for 18 hours in serum-free media as above. The fluorescent positive cells were identified by flow cytometry, isolated and seeded in ECMatrix™invasion chambers (Millipore, Billerica, MA, USA). After 24 hours incubation at 37°C with 5% CO_2_, the number of cells that migrated through the ECM layer and attached to the polycarbonate membrane was quantified spectrophotometerically at 560 nm according to the manufacturer's protocol. The assays were done in multiples and the differences in the values between groups were evaluated by analysis of variance (ANOVA). *P *<0.05 was considered significant.

### *In vitro *stability of DNAzyme

AM9D was incubated in PBS at 37ºC, and an equal amount was removed at various time points and incubated with *MMP9 *mRNA at 37ºC. After a 2-hour incubation the RNA samples were visualized on a 4% urea-polyacrylamide gel. For DNAzyme cellular uptake and stability, MDA-MB-231 cells were cultured on cover-glass slides. Cells were then transfected with 4 µg fluorescently labeled DNAzyme, as described above, fixed with formaldehyde at 24, 48, or 72 hours post transfection and visualized by confocal microscopy. The nucleus was visualized by 4',6-diamidino-2-phenylindole (DAPI)/anti-fade.

### Animals

All animal experiments were conducted following approval by the University of Tennessee Health Science Center Institutional Animal Care and Use Committee (IACUC). Friend virus B-type (FVB)/Nj female mice were obtained from Jackson Laboratory (Bar Harbor, ME, USA) and crossed with PyMT-positive FVB males. The offspring were genotyped by real-time PCR on a Roche LC 480 LightCycler using the following primers and universal probe library (UPL) probe #11 (forward primer: 5' AACCCGAGTTCTCCAACAG 3; reverse primer: 5' TCAGCAAC ACAAGGATTTC 3') to identify MMTV-PyMT-positive females. Female mice were palpated once a week beginning at approximately 4 weeks of age and palpable tumors were measured in two dimensions (longest diameter and shortest width) with digital calipers. Tumor volume was calculated using the formula:

$$\text{Tumor}\;\text{volume}=\;\text{Widt}{\text{h}}^{2}\times \;\text{Length}/2$$

When each transgenic female developed at least three palpable tumors of dimensions of 3 mm × 5 mm, which typically occurred at 8 weeks of age, each tumor was injected intratumorally with either 10 or 25 µg of AM9D or control DNAzyme suspended in PBS in a total volume of 5 µl, using a Hamilton syringe mounted with a PT2, 26G needle. Tumors identified at week 0 were injected once per week for a total of 4 weeks of therapy, and the site of intratumoral injection was varied to ensure that all areas of the tumor were exposed to the AMD9 or control DNAzyme. Palpable mammary tumors that arose after week 1 in other mammary glands of the same mice were left untreated. For each cohort, transgenic females with a combined number of at least nine tumors of comparable size were utilized (AMD9, 25 μg, *n *= 2 mice and 12 mammary tumors and control DNAzyme, *n *= 3 mice and 9 mammary tumors). An independent cohort of animals was also included in tumor endpoint volume studies, in which additional mice were treated with either control DNAzyme (25 µg, *n *= 3 mice and 15 mammary tumors) or AM9D (AM9D, 10 µg, n= 2 mice and 9 mammary tumors; 25 µg, *n *= 2 mice and 9 mammary tumors).

Tumor growth was monitored weekly by caliper measurement. All animals were euthanized one week after the last DNAzyme treatment (typically at 12 weeks of age). At necropsy, tumors were removed, final tumor dimensions were measured by calipers and the tumor wet weight was determined. Tumors were then either flash frozen in liquid nitrogen, or fixed in 4% paraformaldehyde overnight, followed by cryoprotection in 25% sucrose for several days. Cryoprotected tumors were then washed with 0.1% PBS prior to embedding in optimal cutting temperature (OCT) compound and preparation of 8-micron sections.

For analysis of *Mmp9 *mRNA expression levels in tumors, OCT compound-embedded tumor sections were scraped from glass slides of individual control DNAzyme or AM9D-treated tumors to form a pool of tumor material, and total RNA and cDNA was prepared and analyzed by RT-PCR analysis as described above.

### Immunohistochemistry

Mammary tumor vasculature was visualized using rat anti-mouse CD31 antibody (1:50) (BD Biosciences, San Jose, CA, USA) and Alexa Fluor-594 goat anti-rat IgG (H+L) secondary antibody (Invitrogen). Stromal cells (myofibroblasts) were detected using anti-α-smooth muscle actin (α-SMA) antibody at 1:250 dilution (Sigma, St. Louis, MO) and Alexa Fluor 488 goat anti-mouse IgG2a (Invitrogen) secondary antibody at 1:500 dilution. MMP-9 protein was detected using a rabbit anti-mouse MMP-9 antibody at a 1:200 dilution (Santa Cruz Biotechnology, Santa Cruz, CA) followed by Alexa Fluor-594 goat anti-rabbit IgG antibody (1:500). Digital images were captured using a Bio-Rad Confocal Laser Scanning Microscope, using the Lasersharp 2000 software. Image J imaging analysis software was used for measurement of MMP-9, CD31-immunostained endothelial area (EA), and caspase-3-positive cells in the scanned immunohistochemistry (IHC) sections of mammary tumors. According to Chantrain *et al. *[32], compared with the so-called hot spot and the random fields methods, the EA measurement method is more reproducible for quantification of tumor vasculature.

### Statistical analysis

All data are expressed as mean ± SD or standard error (SE). Data were analyzed with SSPS software (SigmaStat version 2.03) using one-way analysis of variance (ANOVA), or Student's *t*-test. Tumor growth over time among three groups was analyzed by two-way ANOVA using Prism software (Graphpad version 4.0b, La Jolla, CA). In all cases, *P*-values <0.05 were considered statistically significant.

## Result*s*

### AM9D treatment specifically reduces MMP-9 production and suppresses the invasive behavior of breast tumor cells *in vitro*

**Figure 1:**
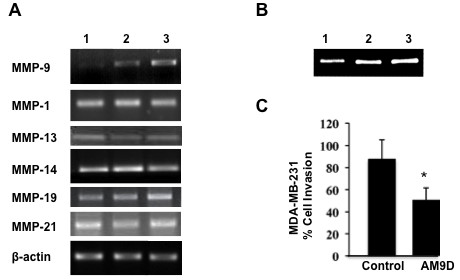
**Effect of AM9D treatment on metalloproteinase (MMP) expression in MDA-MB-231 cells**. (**A**) Expression levels of *MMP9*, *MMP1*, *MMP13*, *MMP14*, *MMP19*, and *MMP21*, and *BACT *(ß-actin) mRNA in MDA-MB-231-transfected cells. MDA-MB-231 cells were transfected with Oregon Green 488-labeled DNAzymes, control DNAzyme or mock transfection reagents as described in Materials and methods. Positively transfected cells were identified by flow cytometry. Total RNA was isolated and *MMP9*, *MMP1*, *MMP13*, *MMP14*, *MMP19 *and *MMP21*, and *BACT *(ß-actin) mRNA were amplified by reverse-transcription (RT)-PCR and the PCR products were subjected to agarose gel and visualized by ethidium bromide staining. Lane 1, AM9D; lane 2, control DNAzyme; lane 3, cells treated with DOTAP (N-[1-(2,3-Dioleoyloxy)]-N,N,N-trimethylammonium propane methylsulfate) transfection reagent only. (**B**) Gelatin zymography of culture media from transfected MDA-MB-231 cells. The cultured media from MDA-MB-231 cells transfected with AM9D (lane 1), control DNAzyme (lane 2), or treated with DOTAP alone (lane 3) were separated on 8% SDS polyacrylamide gel containing 1 mg/ml gelatin. (**C**) Histogram showing the percentage of carcinoma cells invading the ECMatrix™ matrigel matrix after treatment with AM9D compared to cells treated with control DNAzyme. Cells were transfected with Oregon Green 488 labeled-DNAzymes, sorted and cultured in a matrigel matrix invasion chamber as described in Materials and methods. *P <0.05 compared with control (one-way analysis of variance).

The specificity of AM9D toward *MMP9 *mRNA was demonstrated in MDA-MB-231 human breast cancer cells. MDA-MB-231 cells express *MMP1*, *MMP9*, *MMP13*, *MMP14*, *MMP19*, and *MMP21 *(Figure 1A, lane 3). As shown in Figure 1A and 1B, contrary to control DNAzyme (lane 2), AM9D treatment (lane 1) significantly decreased the activity (Figure 1B) and the level of *MMP9 *mRNA (Figure 1A) in MDA-MB-231 cells without having an effect on *MMP1*, *MMP13*, *MMP14*, *MMP19 *or *MMP21 *mRNA levels. Although MMP-2 and -3 have also been reported to contribute to breast tumorigenesis [30], we did not detect *MMP2 *or *MMP3 *mRNA expression in cultured MDA-MB-231 cells. These data demonstrate that the AM9D therapy is specific as it only affects the production of MMP-9 in cells, and that reduction of *MMP9 *mRNA leads to reduction in enzymatic activity, as expected.

The effect of decreased *MMP9 *mRNA expression on the invasive behavior of MDA-MB-231 cells was assessed by transfecting the cells with fluorescently labeled AM9D or control DNAzyme and determining the invasive behavior of the sorted cells using the ECMatrix™invasion chamber. As shown in Figure 1C, the mean invasion potential of MDA-MB-231 decreased by approximately 43% when transfected with AM9D compared to control DNAzyme-treated cells. These data are consistent with the reports of others demonstrating that MMP-9 is one of the key mediators of tumor cell invasion [11,12,33] and supports the idea of the DNAzyme gene-targeted approach for MMP-9 as a breast cancer therapeutic agent.

### MMP-9 is expressed in mammary tumors and the associated stroma in the MMTV-PyMT model

The MMTV-PyMT transgenic mouse model is a widely used pre-clinical model of estrogen and progesterone receptor-negative luminal-like breast cancer with well-defined stages of progression and metastasis to lung [34,35]. More importantly, mammary adenocarcinomas exhibit changes in biomarkers similar to those observed in patients with breast cancer [34,36]. On a pure FVB/Nj strain background, all PyMT-positive females will eventually develop mammary tumors in each of their ten mammary glands, although the time of tumor onset varies among individual glands [35]. The expression patterns of various MMPs in the PyMT model [37-39] are also similar to those observed in patients diagnosed with ductal mammary adenocarcinoma [40]. Therefore, this model was chosen to ascertain the role of AM9D as a pharmacologic inhibitor of MMP-9.

**Figure 2:**
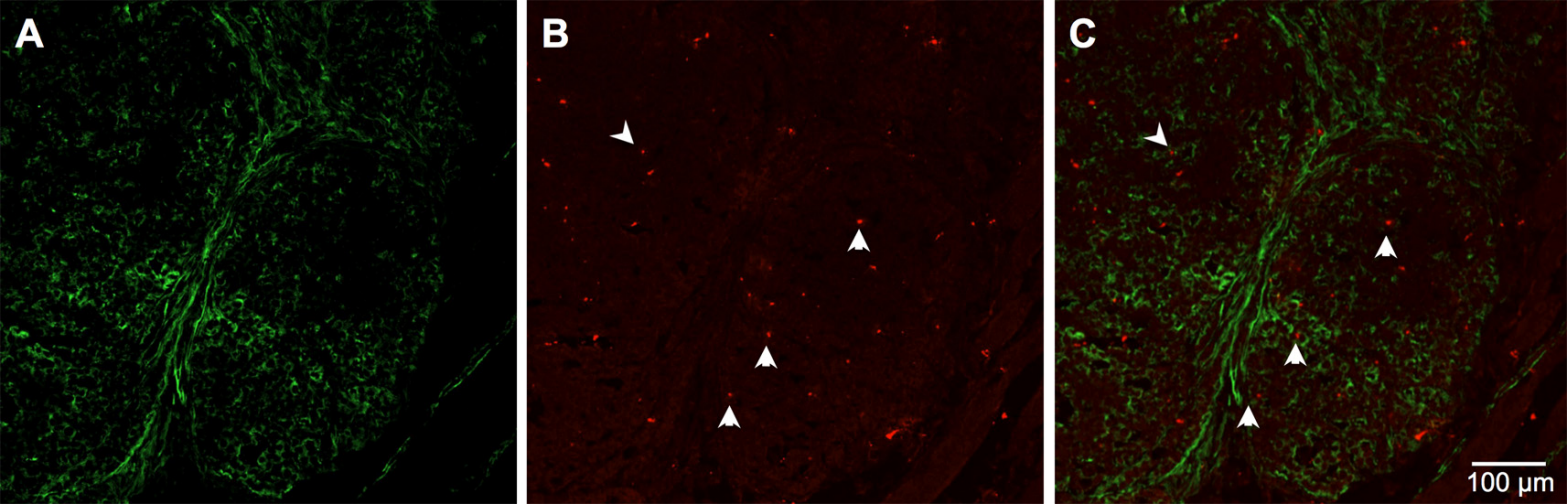
**Immunohistochemical staining for metalloproteinase (MMP)-9 and α-smooth muscle actin (α-SMA) in mammary tumor sections**. Tumors were resected from mice and double stained with antibodies to α-SMA to detect stromal cells **(A) **and MMP-9 **(B)**. When channels were merged **(C)**, these data show that MMP-9 was present in both stromal and tumor cells. Magnification 200×; scale bar is equivalent to 100 µm.

To confirm the presence of MMP-9 protein in late-stage mammary carcinomas, tumors were harvested from MMTV-PyMT transgenic females at 12 weeks of age. Tumor sections were stained with antibodies to both α-SMA, a marker for stromal myofibroblasts, and MMP-9. IHC analysis demonstrated the presence of MMP-9 in the tumor epithelium, including areas highly populated with stromal fibroblasts (Figure 2). It is also likely that MMP-9 is produced by the tumor-associated macrophages that are known to be present in PyMT tumors [41,42].

### DNAzyme is stable *in vitro *and *in vivo *and is present in mammary tumors for at least 14 days post single intratumoral injection

**Figure 3:**
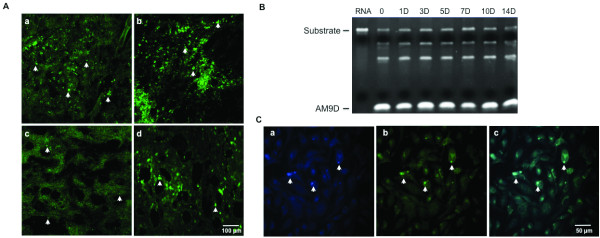
**Stability of DNAzyme in mammary tumors, *in vitro*, and *in vivo***. (**A**) Stability of DNAzyme in mammary tumors. Mammary tumors were injected (as described in Materials and methods) with fluorescently-labeled AM9D and resected at either (**a**) 7 days, (**b) **10 days, or (**c**) 14 days post-injection; (**d**) DNAzyme injected into the 2R tumor of a mouse was found to be distributed to an adjacent, non-injected mammary tumor, 3R, which emerged after intratumoral injections were first initiated. Scale bar is equivalent to 100 µm. (**B) **Urea-polyacrylamide gel electrophoresis of cleaved *MMP*9 RNA by AM9D. AM9D was incubated in PBS at 37ºC for 14 days; an equal amount was removed at days 1, 3, 5, 7, 10, and 14 (1D to 14D, respectively) and incubated with *MMP9 *RNA substrate at 37ºC for 2 hours. The products were then visualized on a 4% urea-polyacrylamide gel. Lane 1, RNA substrate alone; lane 2, AM9D without prior incubation at 37°C (0) cleaved RNA substrate into two fragments. AM9D incubated at 37°C for 1, 3, 5, 7, 10, and 14 days, lanes 3 to 8 respectively, did not lose its catalytic activity toward RNA substrate. (**C**) Stability of AM9D *in vivo*. The MDA-MB-231 cells were transfected with Oregon Green fluorescently labeled AM9D for 72 hours, and fixed and analyzed for the uptake and stability of AM9D molecule in the cells by fluorescent microscopy (400× magnification). (**a**) The nucleus is stained with 4',6-diamidino-2-phenylindole (DAPI) and (**b**) AM9D is shown in green. **(c) **The overlap of AM9D with DAPI staining indicates that AM9D is present in both the cell cytosol and nuclei, as shown by the arrow.

Prior to testing AM9D for its effect on mammary tumor growth, the *in vivo *stability and cellular uptake of naked DNAzyme molecules was examined by intratumorally injecting tumor-bearing MMTV-PyMT transgenic female mice with fluorescently labeled AM9D in PBS. The animals were then sacrificed at 7, 10, and 14 days (Figure 3A, a-c) post AM9D injection, and mammary tumors were harvested, sectioned, and viewed under a fluorescent microscope. As shown in Figure 3A, fluorescently-labeled oligonucleotides could be easily detected in a diffuse pattern within the tumor for up to 14 days (Figure 3A, c). Moreover, AM9D could also be detected in adjacent, non-injected mammary tumors of the same mouse (Figure 3A, d), indicating a wider distribution pattern than might be expected from intratumoral injection. Therefore, the DNAzymes are stable *in vivo *and can efficiently distribute within the injected tumor and to an adjacent non-injected tumor.

To further examine the stability of the DNAzyme in solution and *in vitro*, DNAzyme prepared in PBS was incubated for up to 14 days at 37°C. Aliquots were removed at different time intervals and the amount and activity of DNAzyme remaining over time was determined by applying the DNAzyme to a 6% urea-polyacrylamide gel and measuring its ability to cleave a 760 bp *MMP9 *RNA substrate (Figure 3B). As demonstrated in Figure 3B, DNAzyme oligonucleotides are stable in PBS at 37°C and no significant degradation or loss of enzymatic activity was observed over the 14 day period.

The *in vitro *stability of AM9D was further confirmed by transfecting MDA-MB-231 cells grown on slides with fluorescently labeled AM9D as described above, and visualizing the presence of AM9D in cells by fluorescent microscope at 24, 48 and 72 hours post transfection. As shown in Figure 3C, DNAzyme molecules are present in cells for at least 72 hours post transfection and are located in both the cytosol and the nucleus. The nucleus localization significantly increases the effectiveness of DNAzyme therapy. These data in corroboration with the *in vivo *stability of AM9D administered to mammary tumors of the MMTV-PyMT transgenic mouse (Figure 3A, c-d) demonstrate the retention and potential efficacy of this therapy.

### AM9D treatment reduces final tumor load in the MMTV-PyMT tumor model

**Figure 4:**
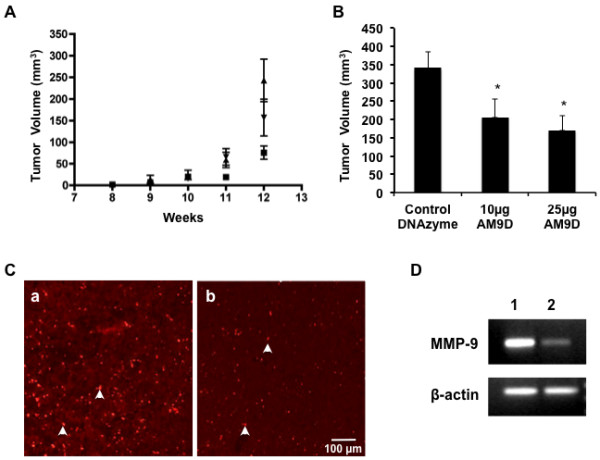
**Effect of AM9D on the rate of tumor growth, final mean tumor volume, and metalloproteinase (MMP)-9 expression**. (**A**) AM9D-treated tumors (■) grew at a slower rate than either untreated tumors (no injections) (▼), or tumors treated with control DNAzyme (▲); at the study endpoint, age 12 weeks, *P *<0.05. (**B**) Weekly intratumoral treatment of transgenic mice with 10 µg (*n *= 9 tumors) or 25 µg AM9D (*n *= 21 tumors) per tumor reduced mean tumor burden by 39.5% or 50.1%, respectively, when compared to tumors treated with control DNAzyme (*n *= 24 tumors) (*P *<0.01 ANOVA). (**C**) Mammary tumors treated with either 25 µg AM9D or control DNAzyme were stained with an MMP-9 antibody. (**a**) Mammary tumors treated with 25 µg control DNAzyme for 4 weeks showed increased MMP-9 staining (arrows) compared to (**b**) mammary tumors treated with AM9D for 4 weeks. Images are shown at 200× magnification; scale bar is equivalent to 100 µm. (**D**) *Mmp9 *mRNA expression levels in control DNAzyme- or AM9D-treated tumors. Total RNA was isolated and pooled from optical cutting temperature (OCT) compound-embedded tumor sections scraped from glass slides of individual control DNAzyme- or AM9D-treated tumors. *Mmp9 *and *Bact *(ß-actin) mRNA were amplified by RT-PCR and the PCR products were subjected to agarose gel and visualized by ethidium bromide staining. Lane 1, AM9D; lane 2, control DNAzyme.

The efficacy of AM9D to reduce breast tumor volume in MMTV-PyMT transgenic mice was tested by directly injecting two concentrations (10 or 25 µg) of AM9D or control DNAzyme into mammary tumors of transgenic females bearing at least three tumors per mouse, each at an early palpable size (approximately 3 mm × 5 mm), once a week for 4 weeks. Tumor palpations were performed weekly to determine changes in tumor volume over time. The growth rate of AM9D-treated tumors (*n *= 12) was slower than both control DNAzyme-treated tumors (*n *= 9) and untreated tumors (*n *= 7) (Figure 4A). This resulted in a significant reduction in the final tumor volume of AM9D-treated compared to control DNAzyme-treated (*P *<0.001) and untreated (*P *<0.01) tumors at age 12 weeks (Figure 4A). In fact, administration of AM9D at 10 µg was sufficient to reduce the size of the tumor by 39.5% (*n *= 9, *P *≤0.01) compared to control (*n *= 24), which increased to 50% when 25 µg of AM9D was utilized (*n *= 21, *P *≤0.01) (Figure 4B).

IHC analysis of the mammary tumors (Figure 4C) confirmed that AM9D treatment successfully downregulated MMP-9 protein expression. As shown in Figure 4C (b), AM9D treatment reduced mean MMP-9 expression by 66 ± 11% as compared to the control DNAzyme treatment (Figure 4C, a). This was further confirmed by the observation that the *Mmp9 *mRNA levels were 77% lower in AM9D-treated tumors compared with those tumors treated with control DNAzyme (Figure 4D). Taken together, these data show that AM9D efficiently decreases MMP-9 expression in tumors, resulting in the observed anti-tumor effects.

### AM9D treatment suppresses angiogenesis and stimulates apoptosis in mammary tumors

**Figure 5:**
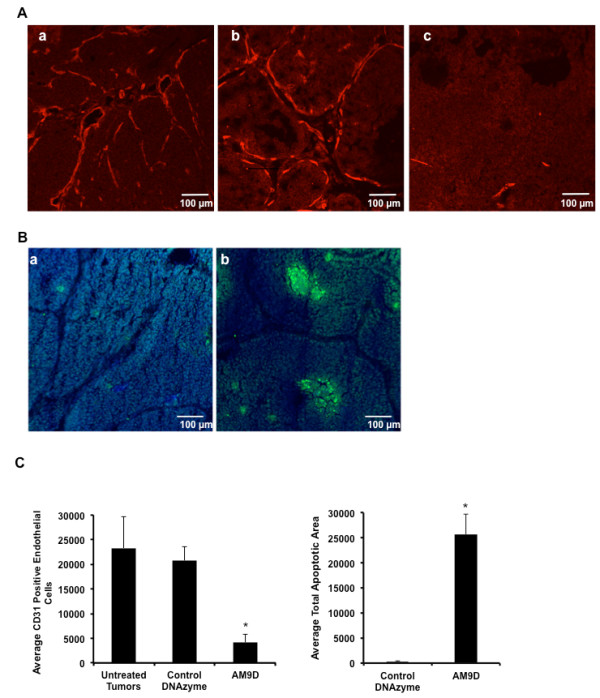
**Angiogenesis and apoptosis immunohistochemical staining of tumors**. (**A**) Staining for CD31 (red) to detect the vasculature in (**a**) untreated mammary tumors and mammary tumors harvested from mice treated with (**c**) 25 µg of AM9D or (**b**) control DNAzyme. Untreated tumors (**a**) and tumors treated with control DNAzyme (**b**) contained more blood vessels than tumors treated with AM9D (**c**). Images are shown at 200× magnification. Scale bar is equivalent to 100 µm. (**B**) Immunohistochemical staining for caspase-3 to indicate apoptosis in mammary tumors harvested from mice treated for 4 weeks with (**a**) control DNAzyme or (**b**) 25 µg AM9D. Very few caspase-3-positive cells (green) were detected in tumors treated with control DNAzyme (**a**) compared to AM9D-treated (**b**) tumors, where large regions stained positive for caspase-3 (green). Scale bar is equivalent to 100 µm. (**C**) Histogram showing the mean CD31-immunostained endothelial area and mean apoptotic area in mammary tumors following treatment with AM9D as compared to untreated tumors and control DNAzyme-treated tumors. *P <0.05 compared with control (one-way analysis of variance and Student's *t*-test).

MMP-9 has been shown to play a role in tumor progression through increase of bioavailability of VEGF and other factors that promote angiogenesis [43]. To determine the mechanism of tumor volume reduction by AM9D, the tumor slices were stained for CD-31 and for activated caspase-3 to assess the effect of AM9D on angiogenesis and apoptosis, respectively. As shown in Figure 5A and 5B, AM9D treatment significantly reduced the number of blood vessels in the tumor as demonstrated by the lack of robust CD-31 immunostaining in the AM9D-treated group (Figure 5A, c) versus untreated (Figure 5A, a) or the control DNAzyme-treated (Figure 5A, b) groups. Moreover, our data also indicate that AM9D potently induces apoptosis in the tumors, as only AM9D-treated tumors contained a large number of caspase-3-positive cells, as shown in Figure 5B (b). Quantitative analysis (Figure 5C) indicated that the number of CD31-positive cells was reduced 5-fold and that the intensity of the apoptotic cells increased 83-fold in tumors treated with AM9D compared to controls, respectively. These data suggest that the simultaneous anti-angiogenic and pro-apoptotic effect of AM9D delays tumor growth over time, and decreases tumor volume at our study endpoint.

## Discussion

In this study, we showed for the first time, that the downregulation of MMP-9 in mammary tumors by a novel anti-MMP-9 DNAzyme molecule results in a significant reduction in final tumor volume in the MMTV-PyMT transgenic mouse model of breast cancer. Downregulation of MMP-9 by AM9D was accompanied by a decrease in MMP-9 expression, decreased angiogenesis and increased apoptosis. Moreover, these effects were accomplished by intratumoral injection of naked DNAzyme without the use of any carriers. AMD9 treatment also reduced the invasive potential of cultured MDA-MB-231 cells *in vitro *(Figure 1C). Together, these data indicate that specific inhibition of MMP-9 expression by DNAzyme has potential as a novel therapeutic modality to decrease the growth and invasion of carcinoma cells in the clinical setting.

It is known that MMP-9 plays a key role in angiogenesis by releasing VEGF [43] and that its downregulation induces apoptosis by stimulating the ERK pathway [17]. Martin *et al. *[44] have demonstrated that tumors developed in MMTV-PyMT MMP-9 wild-type mice are larger in size and are more highly vascular compared to those tumors that developed in MMTV-PyMT MMP-9-null mice. Thus, these data suggest that AM9D treatment affects tumor growth via different pathways, as downregulation of MMP-9 by AM9D inhibited angiogenesis and induced apoptosis (Figure 5) as demonstrated by lack of CD31 staining and the enhanced presence of caspase-3 in AM9D-treated tumors.

Our results are consistent with those of Almholt *et al. *[40] in which the broad-spectrum MMP inhibitor, Galardin/GM6001, significantly reduced primary mammary tumor growth and lung metastasis in the MMTV-PyMT model. However, contrary to broad-spectrum MMP inhibitors, including GM6001, AM9D treatment specifically downregulates MMP-9 without affecting the expression of other members of the MMP family. As demonstrated by the extent of cytoxicity of broad-spectrum MMP inhibitors in prior clinical trials [45-47], total inhibition of MMP is not practical. Various MMPs can exert both pro-tumorigenic and anti-tumorigenic properties [48], and some MMPs are critical for normal physiological processes, such as bone growth and remodeling, ovulation, and wound healing [49]. Further, in comparison with GM6001 [40], the intratumoral injection of AM9D not only reduced the required frequency of therapy, but was also equally efficient in reducing final tumor size. Once weekly, intratumoral injections of 25 µg AM9D (1.25 mg/Kg) was sufficient to reduce the size of these spontaneously developed tumors by 50% as compared to the 51% tumor reduction observed following daily administration of 100 mg/Kg of GM6001. Thus, the high degree of specificity of AM9D for targeting MMP-9, its *in vivo *stability, and the lack of any observed *in vivo *toxicity (Hallett M, Dalal P, Sweatman T, Pourmotabbed T: Naked Anti-Matrix Metalloproteinase-9 DNAzyme Administered Systemically Distributes to All Organs of Healthy and MMTV-PyMT Transgenic Mice and Is Safe; manuscript in review) should enhance the clinical response of solid tumors, including breast tumors, to AM9D treatment, while evading the serious side effects experienced with systemic therapy based on broad-spectrum MMP inhibitors.

The MMTV-PyMT transgenic model limited our ability to assess the efficacy of AM9D on treating spontaneous lung metastasis *in vivo *because not all tumors in each animal grow synchronously, and thus, not all tumors were intratumorally treated with therapy. Therefore, it was not feasible to determine the origin of metastatic cells (from treated or untreated tumors). The efficacy of AM9D in inhibiting lung metastasis is under investigation using a mouse model of metastasis.

## Conclusions

Our results indicate that the downregulation of *MMP*9 mRNA and protein expression with naked anti-MMP-9 DNAzyme is sufficient to reduce mammary tumor burden. We also describe that tumor size reduction is a result of decreased MMP-9 expression, decreased angiogenesis, and increased apoptotic cells in tumors treated with AM9D. These findings suggest specific targeting and downregulation of MMP-9 by AM9D could prove useful as a therapy against breast carcinoma tumor growth and invasion.

## Abbreviations

ANOVA: analysis of variance; α-SMA: alpha smooth muscle actin; AM9D: anti-MMP-9 DNAzyme; bp: base pairs: DAPI: 4',6-diamidino-2-phenylindole; DMEM: Dulbecco's modified Eagles medium; DNAzyme: catalytic oligodeoxynucleotide; EA: endothelial area; ECM: extracellular matrix; Egr-1: early growth response factor-1; ERK: extracellular signal-regulated kinase; FBS: fetal bovine serum; FVB: Friend virus B-type; IHC: immunohistochemistry; MMP: matrix metalloproteinase: MMTV-PyMT: mouse mammary tumor virus-polyoma virus middle T; NF-κB: nuclear factor-κB; OCT: optimal cutting temperature; PBS: phosphate-buffered saline; RT-PCR: reverse transcription-polymerase chain reaction; SE: standard error; VEGFR2: vascular endothelial growth factor receptor 2.

## Competing interests

Tayebeh Pourmotabbed has applied for a patent entitled, *Inhibition of tumour growth and invasion by anti-matrix metalloproteinase DNAzyme*, US Divisional Patent Application Serial number 12/390,628. We have no other competing interests to declare.

## Authors' contributions

MH performed *in vivo *and *in vitro *research, participated in the design and coordination of the study, analysis and interpretation of all results, and drafting of the manuscript. BT performed *in vitro *research and immunohistochemistry. HH participated in the design, execution, acquisition, analysis and interpretation of *in vitro *research. LS provided training for the animal model and wrote the paper. All authors have read and approved the manuscript for publication. TS provided MMTV-PyMT transgenic mice, financial support, and wrote the paper. TP designed research, performed *in vivo *and *in vitro *research, analyzed and interpreted all the data, wrote the paper, and provided financial support.
